# Exploring body mass index and gender-based self-esteem differences in Saudi Arabia

**DOI:** 10.3389/fpubh.2024.1495973

**Published:** 2025-02-05

**Authors:** Maaidah Algamdi

**Affiliations:** Community and Psychiatric Health Nursing Department, Faculty of Nursing, University of Tabuk, Tabuk, Saudi Arabia

**Keywords:** body mass index, self-esteem, obesity, sociodemographic variables, gender-specific pathway

## Abstract

**Background:**

Self-esteem (SE) and obesity have been associated in various studies. This study investigates this relationship among adults in Saudi Arabia. The objectives of this study are to investigate the relationships between SE and body mass index (BMI) and to examine the interactions between sociodemographic-related factors.

**Methods:**

We designed a cross-sectional study using an online survey that included sociodemographics, a BMI measure, and the Rosenberg Self-Esteem Scale.

**Results:**

Levels of SE did not change substantially between the various age groups, as indicated by the Chi-Square test *X^2^* (12, *N* = 332, = 5.278, *p*-value = 0.948). The results for males reveal that there is a variation in the levels of SE across the different BMI categories. This suggests that the BMI categories have a major influence on the levels of SE among males. In both genders, the results indicate a negative association between variables, with a higher BMI being associated with a lower level of SE. The significance of this association stands for both genders (*p*-value <0.001). For males, the association has a greater influence (Estimate = −0.110, *p*-value <0.001) than it does for females (Estimate = −0.099, *p*-value <0.001). In females, the negative link is larger for education (−0.273) and highly impactful (*p*-value <0.001) in comparison to men (Estimate = −0.157, *p*-value <0.001). Higher education levels are associated with a lower BMI (*p*-value = 0.018). For men, the indirect effects show that education (Estimate = 0.0173*) and marital status (Estimate = −0.0405*) significantly influence SE, with other factors mediating these effects. Both genders experience significant and detrimental impacts from BMI on SE, with males experiencing a more pronounced impact. There are considerable disparities in the ways in which these parameters impact SE in both genders, as revealed by the comparisons of the nested models.

**Conclusion:**

There is a negative correlation between BMI and SE in both genders, with a more pronounced impact in men. Gender-specific differences in the relationship between BMI and SE underscore the importance of considering distinct pathways for males and females in future analyses.

## Introduction

Recent years have seen the emergence of obesity as a disease that is both complicated and multifaceted, and it continues to have a broad influence on a worldwide scale. The prevalence of obesity has grown considerably over the last four decades, reaching approximately a third of the world’s population at present (1, 2). By 2040, more than 50% of people will be obese or overweight, and the global pandemic of obesity, known as ‘globesity’, has gained widespread recognition (3–5). Compared to men, women are more likely to be overweight or obese due to the unique physical changes associated with pregnancy and childbirth, as well as the fact that they lead more sedentary lifestyles and are thus more vulnerable to the harmful effects of obesity and overweight (4). Comorbidities, such as type 2 diabetes, certain types of cancer, hypertension, heart failure, stroke, and hypercholesterolemia, have all been linked to obesity (6–8) and have led to increased healthcare expenses (9). The net result is a poor quality of life for those who are struggling with excessive body weight (10).

In addition to these detrimental effects, people who are overweight or obese usually face prejudice and social stigma, which makes the already complex issue much more difficult to deal with (11). Accordingly, obese people typically exhibit lower levels of self-esteem (SE) than non-obese people (12–15), due to society’s negative opinion about them (16). Furthermore, a higher prevalence of negative body image in females compared to males relates to poor SE, as are many psychological problems, including behavioral disorders, melancholy moods, and unpleasant, uncontrollable emotions (17–19). The negative relationship between SE and obesity is well-documented (20, 21), and research in Jordan and Saudi Arabia further supports this inverse correlation (22, 23). Certain studies indicate a causal association, attributing the reason to inadequate diet and exercise (24). Additionally, a study in Sanandaj, Iran demonstrated that a lifestyle training program led to greater weight loss and improved SE, demonstrating the positive impact of weight loss on the mental health of obese individuals (25). In fact, a study in Esfahan, Iran reported significant improvements in SE and body mass index (BMI) following cognitive-behavior therapy, revealing that both lifestyle and cognitive interventions effectively reduced BMI and enhanced the quality of life in obese patients (26). Research has widely demonstrated that BMI, a negative biological component, contributes to negative body image and anxiety about unfavorable appraisal (19, 27–29). Higher levels of SE may also be linked to increased levels of physical activity, which improves weight status (30), as well as healthy eating habits, including a stronger adherence to a healthy diet, such as the Mediterranean diet (31), and a lower consumption of soft beverages. While several studies have found a possible correlation between SE and weight (32–34), no studies have found a definitive link between the two.

Despite the increasing amount of research on the various risk factors for weight gain, such as education, genetics, biology, sociodemographic factors, environment, and behavior (35, 36), the fundamental elements that could lead to an understanding of the interaction between men and women in Saudi Arabia remain unclear. As far as we are aware, there has not been any study looking at the probability of a correlation between weight changes and SE adjustments in a large adult population over time. This study examined SE, BMI, and other sociodemographic and lifestyle factors. With the following study questions, we intend to determine the extent to which there is a relationship between BMI and SE among people in Saudi Arabia: (1) What is the prevalence of obesity and SE among the studied sample? (2) Is there any relationship between the sociodemographic characteristics of the sample and the BMI and SE? What is the relationship between BMI and SE?

## Methodology

### Design

The study used an observational, cross-sectional approach. We collected data from individuals in Saudi Arabia to assess the relationship between BMI, SE, and the participants’ sociodemographic characteristics.

The research required a convenient sample of Saudi Arabian individuals aged 18 and above who possessed literacy skills. The sample size was determined utilizing the following formula: *n* = N * X/ (X + N – 1), where X was defined as Z*α*/2 * p * (1 - p) /MOE^2^. Here, Zα/2 represents the critical value of the normal distribution at α/2 (e.g., at a 95% confidence interval, α = 0.05 and *c* = 1.96), MOE denotes the margin of error, p indicates the sample percentage, N refers to the population size, and n = sample size. Given the indeterminacy of the prevalence percentage, we applied an estimate of 50%. This formula produced a sample size of 384. We collected data over 4 months using an online survey, completing a total of 332 surveys. The dependent variable was SE, while the independent variables included age, gender, marital status, education, occupation, weight, height, smoking, and chronic illness. The cross-sectional design examined the moderating effect of BMI.

### Data collection method

We collected data using an online survey over a four-month period. We distributed the survey using various social media platforms to reach a diverse audience. This approach ensured wide accessibility and convenience for participants, enabling responses from individuals across different locations and demographics.

### Instruments

In this study, we used three instruments to assess the variables: the sample’s sociodemographic characteristics, SE, and BMI. Sociodemographic characteristics included age, gender, marital status, education, occupation, weight, height, smoking, and chronic illness. We assessed self-esteem using the Rosenberg Self-Esteem Scale (RSES) (37), following the original manual’s scoring system that divided the scores into three categories: poor (0–14.9), average (15–25), and high (25.1–30). BMI was calculated according to the following formula: (weight in kilograms/square height) kg/m^2^. We categorize BMI as follows (38): underweight (15–19.9), normal weight (20–24.9), overweight (25–29.9), Class I (30–34.9), Class II (35–39.9), and Class III (≥ 40).

### Data analysis

We used Analysis of Moment Structures (AMOS) for structural equation modelling (SEM) to evaluate the direct and indirect relationships between one or more independent latent variables and dependent variables, providing a more robust analysis than traditional multivariate methods like multiple regression. SEM enables the simultaneous simulation of multiple independent and dependent constructs. We coded the data in an Excel sheet, treated it for missing variables, and then transferred it into the Statistical Package for Social Science (SPSS) software. We performed the analysis using SPSS version 23.0 (IBM, Armonk, NY, USA) at a 95% confidence interval. We checked the data for completeness and correctness. Typically, we use normality tests to verify the normal distribution of a continuous variable. However, categorical variables, not being continuous, cannot conform to a normal distribution. Nominal data represent categories without a specific order or ranking, such as gender, marital status, occupation, etc. Unlike continuous or ordinal data, nominal data do not have a numerical relationship that allows for skewness or kurtosis analysis (39–42). As an example, it’s possible that answers in datasets like 1, 2, 3, and 4 are three times more likely to be in a higher category than the lowest category. We might treat them similarly to 1 or 2, assuming equal distances, which could lead to erroneous conclusions (43). Therefore, normality does not apply to nominal data in the same way as it does to interval or ratio data (44).

### Ethical consideration

We obtained ethical approval # UT-120-15-2020 from the University of Tabuk Local Research Ethic Committee, attached informed consent to the beginning of the questionnaire, and allowed anyone who agreed to participate to fill it out without needing to sign a name or phone number. Participation in the study is entirely voluntary, and we maintain anonymity and confidentiality throughout all stages of the study. The principal investigator provided the participants with complete contact information for informed consent.

## Descriptive statistics of demographic variables

Table 1 provides a comprehensive overview of the distribution of various demographic factors among the research participants (*N* = 332). The variables consist of gender, marital status, education, employment, smoking status, chronic illness status (ChronicDx), BMI categories, and the RSES scores. The variable “Gender” categorizes the distribution of participants based on their gender in the research sample. Of the 332 participants, 62.35%, or 207 individuals, belong to the male gender. This indicates that a substantial majority of the individuals included in the study are male, underscoring a potential disparity in gender representation within the sample. Conversely, the data reveals that 125 individuals, comprising 37.65% of the entire sample, are female. The gender distribution in this study gives important contextual information on the representation of each gender within Saudi religious and cultural backgrounds. This information might have an impact on the generalizability and applicability of the study’s findings to larger groups. The chi-square test produced a significant outcome (Chi-Square = 20.253, df = 1, *p*-value = <0.001), signifying a statistically significant difference in gender distribution within the sample. The chi-square test results (Chi-Square = 240.072, df = 3, *p*-value = <0.001) demonstrate a considerable difference in the distribution of marital status among the research participants. There exists a substantial disparity in levels of education, occupational distribution among participants, smoking status, prevalence of chronic illness, and classifications of participants’ BMI. The RSES figures indicate that most participants had medium self-esteem, whereas a lesser percentage demonstrated negative SE.

**Table 1:** Demographic and health variables with their chi-square tests.

Variable	Response	*n*	%	Chi-square	df	*P*-value
Age groups	18–25	168	50.6	431.08	6	<0.001
	26–33	68	20.5			
	34–41	45	13.6			
	42–49	33	9.9			
	50–57	15	4.5			
	58–65	2	0.6			
	66–73	1	0.3			
	Total	332	100.0			
Gender	Male	207	62.35	20.253	1	<0.001
	Female	125	37.65			
	Total	332	100			
Marital status	Single	152	45.78	240.072	3	<0.001
	Married	155	46.69			
	Divorced	17	5.12			
	Widow	8	2.41			
	Total	332	100			
Education	Elementary	7	2.11	403.169	6	<0.001
	Intermediate	8	2.41			
	High School	78	23.49			
	Diploma	25	7.53			
	Bachelor	162	48.80			
	Master	11	3.31			
	Doctorate	41	12.35			
	Total	332	100			
Occupation	Employed	153	46.08	147.783	3	<0.001
	Unemployed	62	18.67			
	Retired	5	1.51			
	Student	112	33.73			
	Total	332	100			
Smoking	Yes	87	26.20	75.193	1	<0.001
	No	245	73.80			
	Total	332	100			
Chronic disease	Yes	54	16.27	151.133	1	<0.001
	No	278	83.73			
	Total	332	100			
BMI	Underweight	48	14.46	244.867	5	<0.001
	Normal weight	154	46.39			
	Overweight	63	18.98			
	Class I	39	11.75			
	Class II	17	5.12			
	Class III	11	3.31			
	Total	332	100			
Rosenberg Self Esteem Scale	Poor	22	6.63	222.223	2	<0.001
	Average	235	70.78			
	High	75	22.59			
	Total	332	100			

df, degrees of freedom, *P*-value, Probability value, A small *P*-Value (≤ 0.05) suggests strong evidence against the null hypothesis.

## Self-esteem scale by demographic and health variables

**Figure 1:**
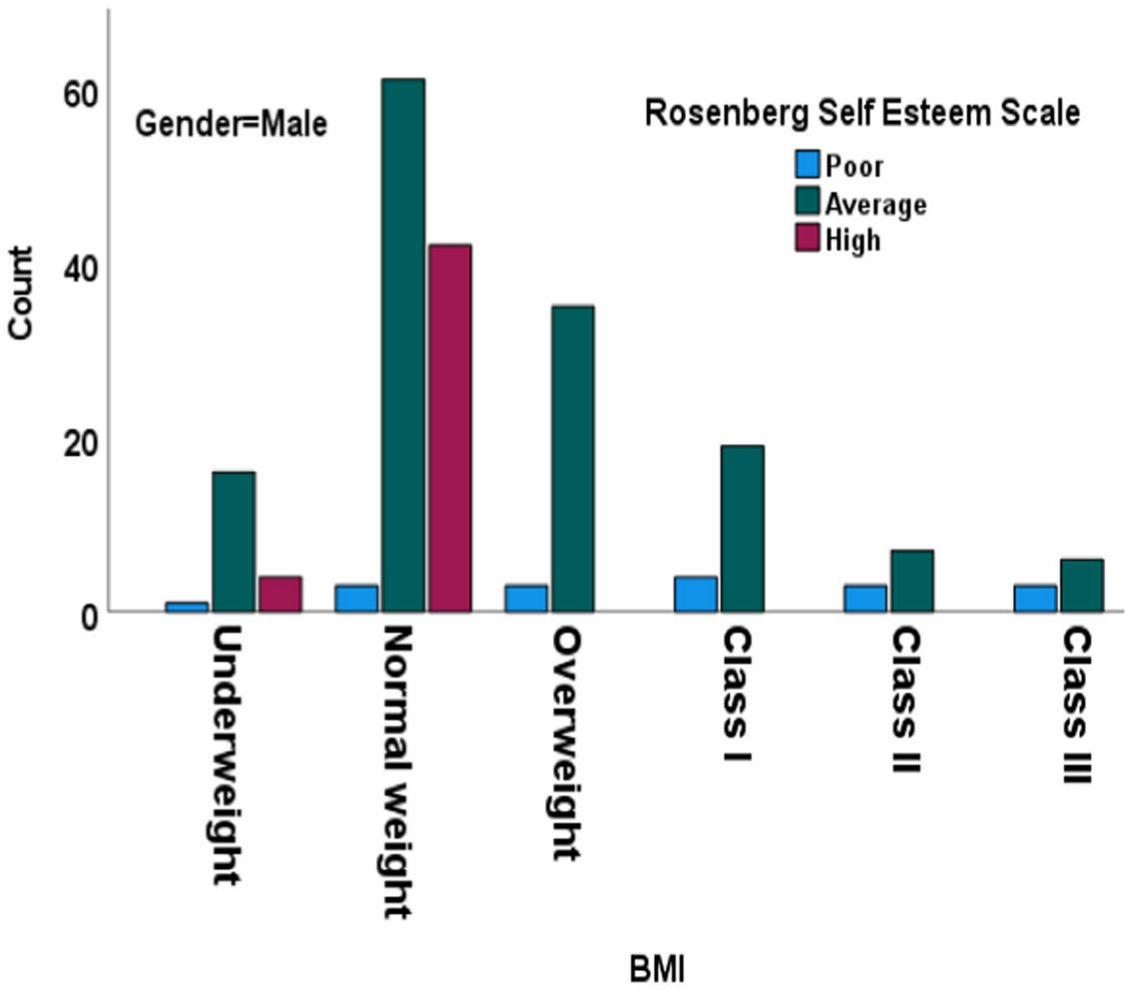
Chi-square test results for Rosenberg Self-Esteem Scale across BMI categories in males. BMI: body mass index. Df, degree of freedom.

Table 2 presents the results of the RSES across various demographic and health variables, using chi-square tests for independence. The age groups ranged from 18 to 73 years. SE levels did not vary significantly across the different age groups (X^2^ [12, *N* = 332] = 5.278, *p*-value = 0.948). Both male and female participants had similar distributions of SE levels. No significant relationship between gender and SE levels was observed (X^2^ [2, *N* = 332] = 2.235, *p*-value = 0.327). The participants’ marital status significantly influenced SE levels by associating different marital statuses with varying levels of SE. We found a significant relationship between education level and SE, with higher educational attainment significantly influencing different levels of SE, suggesting that education significantly influences SE. The distribution of SE varied significantly across occupational statuses, which highlighted the impact of occupation on SE. Furthermore, the SE levels of smokers and non-smokers varied significantly, and the presence or absence of chronic diseases significantly correlated with SE, indicating that individuals with chronic diseases had different SE levels compared to those without. Figure 1 for males shows the distribution of SE levels across different BMI categories.

**Table 2:** SE scale by demographic and health variables.

SE	Variables								Chi-square	df	P-Value
**Age groups of participants**											
	**18–25**	**26–33**	**34–41**	**42–49**	**50–57**	**58–65**	**66–73**	**Total**	5.278	12	0.948
Poor	12	3	4	3	0	0	0	22			
Average	121	46	30	24	11	2	1	235			
High	35	19	11	6	4	0	0	75			
Total	168	68	45	33	15	2	1	332			
**Gender**											
	**Male**	**Female**						**Total**	2.235	2	0.327
Poor	17	5	-	-	-	-	-	22			
Average	144	91	-	-	-	-	-	235			
High	46	29	-	-	-	-	-	75			
Total	207	125	-	-	-	-	-	332			
**Marital status**											
	**Single**	**Married**	**Divorced**	**Widow**			**Total**		242.096	6	<0.001
Poor	1	2	13	6	-	-	22				
Average	134	95	4	2	-	-	235				
High	17	58	0	0	-	-	75				
Total	152	155	17	8	-	-	332				
**Education**											
	**Elementary**	**Intermediate**	**High School**	**Diploma**	**Bachelor**	**Master**	**Doctorate**	**Total**	147.594	12	<0.001
Poor	0	0	22	0	0	0	0	22			
Average	7	8	56	25	116	9	14	235			
High	0	0	0	0	46	2	27	75			
Total	7	8	78	25	162	11	41	332			
**Occupation**											
	**Employed**	**Unemployed**	**Retired**	**Student**			**Total**		67.434	6	<0.001
Poor	9	4	0	9	-	-	22				
Average	79	58	5	93	-	-	235				
High	65	0	0	10	-	-	75				
	153	62	5	112	-	-	332				
**Smoking**											
	**Yes**	**No**					**Total**		39.687	2	<0.001
Poor	18	4	-	-	-	-	22				
Average	57	178	-	-	-	-	235				
High	12	63	-	-	-	-	75				
	87	245	-	-	-	-	332				
**Chronic disease**											
	**Yes**	**No**					**Total**		65.513	2	<0.001
Poor	17	5	-	-	-	-	22				
Average	31	204	-	-	-	-	235				
High	6	69	-	-	-	-	75				
Total	54	278	-	-	-	-	332				

df, degrees of freedom, *P*-value, Probability value, A small *P*-Value (≤ 0.05) suggests strong evidence against the null hypothesis.

**Figure 2:**
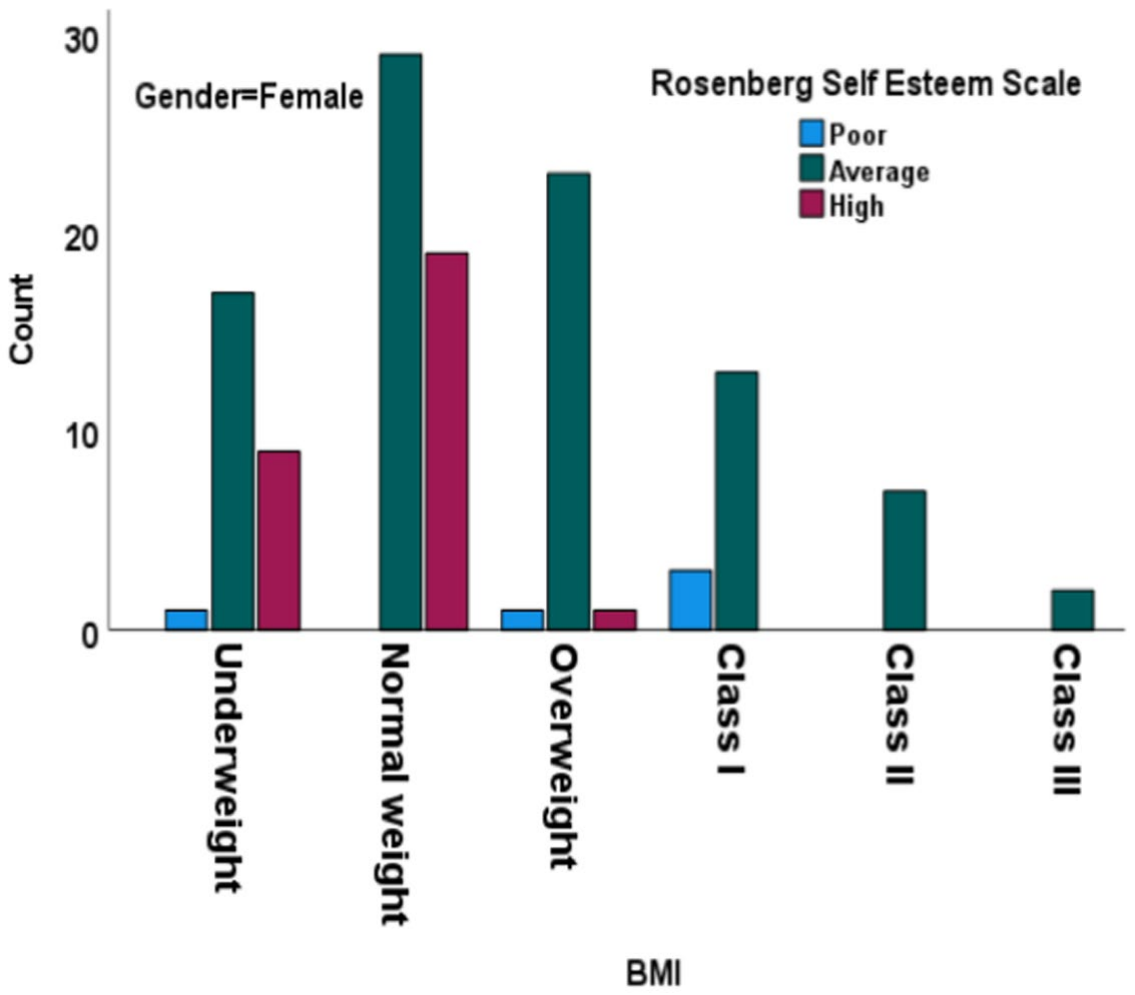
Chi-square test results for Rosenberg Self-Esteem Scale across BMI categories in females. BMI: body mass index. Df, degree of freedom.

We categorized SE levels into three categories: poor, average, and high. Most underweight males fell into the average SE category (16), with fewer in the high (4) and poor (1) categories. Only three members of the normal-weight group fell into the poor category, compared to a higher number of members in the average (61) and high (42) categories. This suggests a positive correlation between normal weight and higher SE. Notably, there were no members from the Overweight, Class I Obesity, Class II Obesity, and Class III Obesity groups who were in the high SE category. Furthermore, the chi-square test for males showed a significant difference in SE levels across BMI categories, indicating that BMI categories significantly impact SE levels among males. Most normal-weight females (Figure 2) were in the average (29) and high (19) categories, with none in the poor category, suggesting a strong correlation between normal weight and higher SE (*p*-value <0.001). Both males and females had a majority in the average SE category, but females had a higher count in the high SE category compared to males. Both genders exhibited higher SE among members of the normal-weight category, with males having a slightly higher count in the high SE category. The chi-square test results reinforce the observation that BMI categories significantly impact SE levels for both genders, with normal-weight individuals generally exhibiting higher SE compared to those in higher BMI categories. This underscores the relationship between BMI and SE and highlights the psychological implications of weight categories on self-perception and confidence levels in both males and females.

## Model fit diagnostics for unconstrained and constrained model

Table 3 contrasts the model fit indices for the unconstrained and constrained (male vs. female groups) models. Table 3 contrasts the reported indices. The indices include the Tucker-Lewis index (TLI), comparative fit index (CFI), root mean square error of approximation (RMSEA), root mean square residual (RMR), normed fit index (NFI), relative fit index (FRI), incremental fit index (IFI), goodness of fit index (GFI), adjusted goodness of fit index (AGFI), and chi-square value (CMIN). The male vs. female model had a CMIN/DF ratio of 2.1243, whereas the unconstrained model had a CMIN/DF ratio of 2.1388. Furthermore, the unconstrained model had an RMR of 0.0197, a GFI of 0.9957, and an AGFI of 0.9105 in the RMR and GFI sections. The RMR, GFI, and AGFI for the male vs. female models were 0.0449, 0.9775, and 0.9141, respectively. The Baseline Comparisons section displays the fit indices for both models—NFI, RFI, IFI, TLI, and CFI. Each of these indices showed a strong match. The unconstrained model had an RMSEA of 0.0587, while the male vs. female model had an RMSEA of 0.0584. Thus, all metrics followed the threshold values (45).

**Table 3:** Model fit summary of unconstrained vs constrained (Male vs Female) models.

CMIN					
Model	NPAR	CMIN	DF	p-value	CMIN/DF
Unconstrained	40	4.2777	2	0.1178	2.1388
Male vs. Female	31	23.3675	11	0.0157	2.1243
RMR, GFI					
Model	RMR	GFI	AGFI		
Unconstrained	0.0197	0.9957	0.9105		
Male vs. Female	0.0449	0.9775	0.9141		
Baseline comparisons					
Model	NFI	RFI	IFI	TLI	CFI
	Delta1	rho1	Delta2	rho2	
Unconstrained	0.9889	0.8342	0.9941	0.9043	0.9936
Male vs. Female	0.9396	0.8353	0.9671	0.9055	0.9654
RMSEA					
Model	RMSEA	LO 90	HI 90	PCLOSE	
Unconstrained	0.0587	0	0.1372	0.3242	
Male vs. Female	0.0584	0.0244	0.0914	0.3002	

CMIN, The chi-square value; NPAR, Number of Parameters; CMIN/DF, Chi-square divided by degrees of freedom; RMR, Root Mean Residual; GFI, Goodness-of-Fit Index; AGFI, Adjusted GFI; NFI, Normed Fit Index; CFI, Comparative Fit Index; RFI, Relative Fit Index; TLI, Tucker-Lewis Index; IFI, Incremental Fit Index; RMSEA, Root Mean Square Error of Approximation; LO 90/HI 90, The 90% confidence interval for RMSEA; PCLOSE, Tests whether RMSEA is close to 0.

## Impact analysis

The analysis indicates that for both genders, a higher BMI is associated with lower SE, demonstrating a negative relationship (Table 4). This relationship is statistically significant for both males and females (*p*-value <0.001). However, the impact of BMI on SE is slightly more pronounced for males, with an estimate of −0.110, compared to an estimate of −0.099 for females. While both genders link increased BMI to decreased SE, the effect is marginally stronger in males, suggesting that BMI may play a more critical role in influencing SE in men than in women. For both genders, a negative relationship suggests that higher BMI decreases SE. The relationship is more impactful for males (Estimate = −0.110, *p*-value <0.001) than for females (Estimate = −0.099, *p*-value <0.001).

**Table 4:** Gender-based differences in regression paths and nested model comparisons.

Regression paths	Gender	Estimate	S.E.	C.R.	*p*-value
SE ← BMI	Male	−0.110	0.024	−4.628	***
	Female	−0.099	0.029	−3.315	***
BMI ← Marital Status	Male	0.367	0.130	2.821	***
	Female	0.310	0.177	1.750	0.080
BMI ← Education	Male	−0.157	0.067	−2.356	***
	Female	−0.273	0.068	−3.994	***
BMI ← Occupation	Male	0.049	0.066	0.735	0.462
	Female	−0.186	0.091	−2.043	***
BMI ← Smoking	Male	−0.139	0.172	−0.806	0.420
	Female	−0.164	0.433	−0.378	0.704
SE ← Marital Status	Male	−0.148	0.045	−3.265	***
	Female	−0.003	0.059	−0.050	0.959
SE ← Education	Male	0.151	0.023	6.539	***
	Female	0.098	0.024	4.081	***
SE ← Occupation	Male	−0.099	0.023	−4.392	***
	Female	−0.116	0.031	−3.770	***
SE ← Smoking	Male	0.155	0.060	2.632	***
	Female	0.611	0.144	4.235	***

Model	DF	CMIN	*p*-value
Nested model comparisons			
Structural weights	9	19.089	***
Structural residuals	20	104.853	***
Male vs. Female	9	19.089	***

BMI, Body Mass Index. S.E, Standard Error. C.R, Critical Ratio. ∗*p* ≤ 0.05. ** *p* ≤ 0.01. *** *p* ≤ 0.001.

For males, a positive relationship (Estimate = 0.367) indicates that being married or having a certain marital status increases BMI (*p*-value = 0.004). For females, the relationship is also positive (Estimate = 0.3095), but not statistically significant (*p*-value = 0.080). For males, a negative relationship (Estimate = −0.157) suggests that higher education levels are associated with lower BMI (*p*-value = 0.018). For females, the negative relationship is stronger (estimate = −0.273) and highly impactful (*p*-value <0.001). For females, a negative relationship (Estimate = −0.186) suggests certain occupations are associated with lower BMI (*p*-value = 0.040). For males, the relationship is not significant (Estimate = 0.049, *p*-value = 0.462). Regarding smoking, the relationship is not significant for both males (Estimate = −0.139, p-value = 0.420) and females (Estimate = −0.164, *p*-value = 0.704). For males, a negative relationship (Estimate = −0.148) indicates that being married or having a certain marital status decreases SE (*p*-value = 0.001). For females, the relationship is not significant (Estimate = −0.003, *p*-value = 0.959).

For both genders, a positive relationship indicates that higher education levels increase SE. The relationship is highly impactful for males (Estimate = 0.151, *p*-value <0.001) than females (Estimate = 0.098, *p*-value <0.001). For both genders, a negative relationship suggests that certain occupations decrease SE. The relationship is significant both for males (Estimate = −0.099, *p*-value <0.001) and females (Estimate = −0.116, *p*-value <0.001). For both genders, a positive relationship indicates that smoking increases SE. The relationship is significant for males (Estimate = 0.155, *p*-value = 0.008) and highly significant for females (Estimate = 0.611, *p*-value <0.001). For both genders, a positive relationship indicates that smoking increases SE. The relationship is stronger for females (Estimate = 0.611, *p*-value <0.001) than for males (Estimate = 0.155, *p*-value = 0.008). The nested model comparisons indicate significant differences in the structural weights and residuals between the male and female models. The chi-square values (CMIN) for structural weights and the overall model comparison are significant (*p*-value <0.05), which means that gender seems to change how the predictors and outcomes are related. Thus, higher education and smoking are positively associated with SE for both genders, while a higher BMI is negatively associated with it. The impact of marital status and occupation on BMI and SE varies between males and females.

## Effect evaluation

### Total effects

For males (Table 5), smoking has a positive total effect on SE (Estimate = 0.1702*), while education (Estimate = 0.1684*) and marital status (Estimate = −0.1885*) also significantly influence SE. However, BMI negatively impacts SE (Estimate = −0.1104*). For females, smoking has a much larger positive total effect on SE (Estimate = 0.6279*) compared to males. Education (Estimate = 0.1259*) also has a positive total effect on SE, while occupation (Estimate = −0.0981 ns) and marital status (Estimate = −0.0337 ns) are not significant. BMI negatively impacts SE (Estimate = −0.0991*).

**Table 5:** Gender-based breakdown of total, direct, and indirect effects of BMI on SE.

	Smoking	Occupation	Education	Marital status	BMI
Male					
Total effects					
BMI	−0.1384*	0.0485 ns	−0.1569*	0.3667*	0.000
SE	0.1702*	−0.1047*	0.1684*	−0.1885*	−0.1104*
Direct effects					
BMI	−0.1384*	.0485 ns	−0.1569*	0.3667*	0.000
SE	0.1549*	−0.0993	0.1511*	−0.1480*	−0.1104*
Indirect effects					
BMI	0.000	0.000	0.000	0.000	0.000
SE	0.0153 ns	−0.0054 ns	0.0173*	−0.0405*	0.000
Female					
Total effects					
BMI	−0.1640*	−0.1861*	−0.2733*	0.3095*	0.000
SE	0.6279*	-.0981 ns	0.1259*	−0.0337 ns	−0.0991*
Direct effects					
BMI	−0.164	−0.1861*	−0.2733*	0.3095*	0.000
SE	0.6116*	−0.1166*	0.0989 ns	−0.0030 ns	−0.0991*
Indirect effects					
BMI	0.000	0.000	0.000	0.000	0.000
SE	0.0163 ns	0.0184 ns	0.0271*	−0.0307*	0.000

BMI, Body Mass Index. S.E, Standard Error. C.R, Critical Ratio. ∗*p* ≤ 0.05. ** *p* ≤ 0.01. *** *p* ≤ 0.001.

### Direct effects

For males, the direct effects show that smoking (Estimate = 0.1549*) positively impacts SE, while education (Estimate = 0.1511*) and marital status (Estimate = −0.1480*) are significant influencers. BMI continues to have a negative direct effect on SE (Estimate = −0.1104*). For females, smoking has a strong positive direct effect on SE (Estimate = 0.6116*), while occupation (Estimate = −0.1166*) is negatively significant. Education (Estimate = 0.0989 ns) and marital status (Estimate = −0.0030 ns) do not have significant direct effects. BMI negatively impacts SE (Estimate = −0.0991*).

### Indirect effects

**Figure 3:**
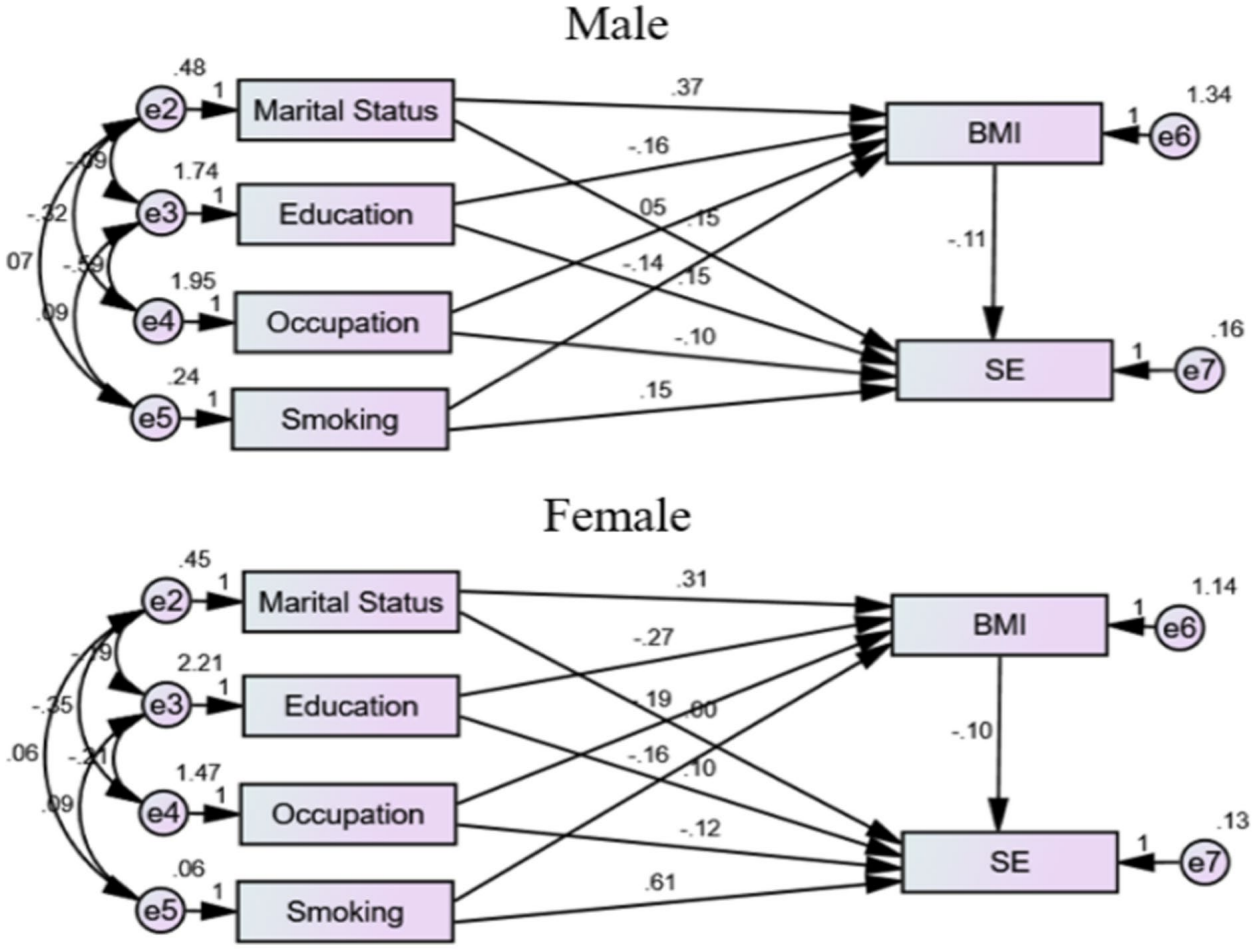
Gender-specific demographic impacts on SE. BMI: body mass index. SE: self-esteem.

For males, indirect effects show that education (Estimate = 0.0173*) and marital status (Estimate = −0.0405*) have significant impacts on SE, mediated by other variables. Smoking and occupation do not have significant indirect effects on SE. For females, education (Estimate = 0.0271*) and marital status (Estimate = −0.0307*) have significant indirect effects on SE, while smoking and occupation do not have significant indirect effects. The analysis indicates that both the total and direct effects of BMI on SE are negative and significant for both genders, though the impact is slightly more pronounced for men. Smoking has a significant positive impact on SE for both genders, with a stronger effect in females. Education positively influences SE in both genders, while occupation and marital status show varying impacts. The nested model comparisons reveal significant differences in how these factors influence SE between males and females, emphasizing the importance of considering gender-specific pathways when understanding the relationship between BMI and SE. Figure 3 visualizes the gender-specific demographic impacts on SE.

## Discussion

This study explored SE levels across various demographic, socioeconomic, and health-related factors. Age ranged from 18 to 73 years, with SE levels remaining consistent across age groups, suggesting stability of SE throughout adulthood. Research on SE stability across adulthood indicates fluctuations depending on life stages. According to Trzesniewski et al. (46) SE stability is quite low during childhood, improves throughout adolescence and early adulthood, and then starts to decline during midlife and older age. Orth et al. (47) observed that SE tends to increase from adolescence to middle adulthood, peaking around ages 50 to 60, before declining into old age. Gender analysis revealed similar SE distributions for males and females, with no significant associations, supporting the idea that gender may not critically influence SE (25). Marital status emerged as a significant factor, with different marital statuses linked to varying SE levels, possibly reflecting the social and emotional dimensions of relationships attributed to the emotional support and social validation inherent in marital relationships (48). Education level showed a strong positive correlation with SE, emphasizing its role in enhancing SE and societal standing, which is in line with a study that found that SE positively predicted academic engagement, which is closely linked to educational attainment (36). Similarly, occupational status significantly influences SE; individuals who are employed tend to report higher SE compared to those who are unemployed; this is due to the positive identity and social validation that employment provides. Employment reinforces worker identity, enhancing self-confidence as a component of SE (49), highlighting the psychological benefits of professional engagement.

Health factors also played a role, with smokers exhibiting distinct SE levels compared to non-smokers. However, studies revealed that smokers often have lower SE, which may influence their smoking behaviors. For instance, Barros et al. discovered that smokers exhibited lower levels of mindfulness and subjective well-being, closely associated with SE, in comparison to non-smokers (50). Additionally, Carter and Byrne reported that people with low SE, particularly in areas such as academics and relationships with parents, were more likely to engage in smoking compared to their peers with higher SE (51). The significant positive correlation between smoking and SE illustrates a paradoxical trend, suggesting that individuals may view smoking as a socially empowering or stress-relieving activity, potentially boosting their self-confidence. The relationship between smoking and SE is complex and varies across different studies. While many studies have found a negative association between smoking and SE, some research suggests that, in certain contexts, smoking may be associated with higher SE, particularly among females. For instance, a Tabriz, Iran study revealed that SE had no conceptual effect on smoking among males. However, the study found that smoking among females decreased with decreasing SE, suggesting that higher SE may be associated with increased smoking behavior among females (52). Additionally, chronic disease presence significantly impacted SE, indicating the influence of physical health on psychological well-being. Ji et al. found that middle-aged and older patients with chronic illnesses exhibited lower SE, which adversely affected their quality of life (53).

The study’s findings for both genders indicate a negative association between a greater BMI and a lower level of SE. The statistical significance of this association was high (*p*-value <0.001) for both males and females. Male BMI had a greater effect on SE than female BMI, with an estimate of −0.110 versus −0.099. Because of this, it appears that, although an increasing BMI is associated with a drop in SE for both males and females, the impact is somewhat stronger in males. This shows that BMI may play a more significant role in determining SE in males than in females.

An existing study showed a larger negative link between BMI and body image among individuals with lower levels of SE (54). These findings are consistent with the results of the current study, which highlight the considerable influence that BMI has on one’s sense of self-worth. There is a negative correlation between a higher BMI and a decrease in the RSES scores for both genders.

In a different study, researchers surveyed 410 teenagers, 51.2% of whom were female and 55.1% of whom were between the ages of 13 and 15 (15). Girls showed a considerably lower SE score than boys did when it came to the categories of obesity and overweight. However, in the overweight group, there was no significant relationship between SE scores based on BMI categories and eating habits, particularly with regard to eating meals with family. This was the case despite the fact that females had a lower mean SE score compared to boys. These findings are consistent with the results of the current study, which highlight the subtle distinctions in the ways that gender and BMI categories affect SE.

One study (23) found that the prevalence of poor SE among Saudi undergraduate women was just 6.1%. However, this percentage was much higher (9.8%) among individuals who were overweight or obese. This conclusion is also consistent with the findings of the current study, which highlight the detrimental effect that a greater BMI has on the SE of females.

Using path analyses to test for mediation, Skorek et al. (55) discovered that self-worth mediated the association between the three personality qualities and body esteem in both men and women. This was the case regardless of whether the individuals were male or female. We found a connection between higher degrees of extraversion, emotional stability, and conscientiousness and higher levels of SE, which in turn led to higher levels of body esteem. However, the model’s incorporation of SE found the associations between personality characteristics and body esteem to be insignificant, indicating the presence of complete mediation. This study lends credence to the notion that SE plays a significant part in mediating the relationship between BMI and general contentment with one’s body image.

In another study, Satwik et al. (56) discovered that factors such as education level, BMI, social support system, diagnosis of mental illness, and perception of menopause were significant predictors of negative body image. The frequency of negative body image among women in their middle years was 17.4%, and this variable had a high correlation with both low SE and mood disorders. Factors such as having a lower education status, a higher BMI, a negative impression of menopause, a weak social support network, and a history of mental health diagnosis enhanced the likelihood of having a bad body image among middle-aged women. Likewise, according to the findings of this study, BMI is one of the numerous essential variables that contribute to body image and SE.

Among pregnant Saudi women, Ghamri et al. (57) discovered a significant positive link between SE and the degree to which they were satisfied with their bodies. The study specifically demonstrated a variable degree of correlation between body image satisfaction, SE, and socioeconomic characteristics, including level of education and income, smoking, and psychiatric and medical comorbidities. Furthermore, the study revealed that the respondents experienced higher levels of Self-Esteem (SE) during the initial weeks of their pregnancies. These studies reveal a strong relationship between body satisfaction and SE, with various elements, including BMI, having an impact.

In their study, Shahzadi and Rasheed investigated the association between gender and SE in obese young girls and boys in Punjab, Pakistan (58). Specifically, they were interested in determining the relationship between body image, BMI, body form dissatisfaction, and body dimensions. In this case, there was a significant correlation between gender, BMI, body shape dissatisfaction, and SE. However, the researchers discovered that there were significant variations between girls and boys in terms of their SE and body image. In comparison to males, girls exhibited significantly higher mean scores for body image dissatisfaction, which resulted in a decrease in their individual SE. This study recommends using the media to increase awareness about the importance of maintaining a positive body image to protect and enhance individuals’ mental and physical health.

Another study conducted by Gómez-Díaz et al. (59) with a total of 55 participants, consisting of 25 males and 30 females, revealed a weight loss of 11 kilograms for females and 16.3 kilograms for males. The study revealed an increase in positive body image perception by 65.2% for women and 76.1% for males, respectively. In addition, there was a rise of 51.4% in the level of SE among women and a rise of 60.3% among males. According to these findings, both men and women can experience considerable changes in their perceptions of their bodies and their levels of SE as a result of weight loss; however, males showed slightly greater benefits.

The present study provides evidence that supports the findings of previous research by indicating that there is a strong negative link between BMI and SE for both males and females. However, the impact was slightly more pronounced in men, indicating the need to tailor interventions aimed at enhancing SE to the specific gender of the target audience. One limitation of the current study is its cross-sectional design and relatively small sample size, which could potentially limit the study’s generalizability. Also, self-reported online surveys might be a potential source of bias. In addition to the cultural and religious nature of society, Saudi Arabia could play a confounding role in the relationship between the studied variables. When conducting research in the future, it will be important to continue investigating these gender disparities and to take into account additional characteristics, such as socioeconomic position, psychiatric comorbidities, and cultural and social factors that have the potential to alter the association between BMI and SE. For people with a higher BMI, it is possible to establish more successful ways to improve their SE and general well-being if they have better knowledge of these complicated relationships. The study’s findings are critical for understanding the participants’ general health and psychological well-being, and they can help inspire future statistical studies to investigate potential relationships and impacts between BMI, SE, and other variables in future studies. Variations in SE across BMI categories for males point to potential connections between physical health and SE, warranting further study. These findings emphasize the multifaceted nature of SE and the significant influence of socio-economic, educational, occupational, and health-related factors. The intricate interplay between socio-economic factors, cultural norms, and personal coping strategies underscores the need for targeted interventions that address these fundamental dynamics, promoting healthier lifestyle choices and enhancing SE through constructive and non-harmful methods.

## Conclusion

While the impact was marginally stronger in men, the results showed that there was a statistically significant negative relationship between BMI and SE for both sexes. This indicates that both men and women experience a decline in SE when their BMI rises, with men feeling the effects to a somewhat greater extent. A man’s BMI increases with marriage or a specific marital status, while a lower BMI is associated with more education. Higher education levels and specific vocations are associated with a lower BMI in women. Neither gender showed a statistically significant relationship between smoking and BMI. Marriage lowered SE in men, but had no effect on women. Higher levels of education positively correlated with SE for both genders, with men experiencing a more pronounced effect. Some jobs reduced SE for both genders, but women felt the impact more acutely. Although the effect was larger in women, smoking had a favorable impact on SE in both genders.

### Implications

- To successfully treat SE concerns connected to body weight, therapies that target specific genders may be required, as the negative influence of BMI on SE is more noticeable in men. When it comes to enhancing SE, it may be more useful for men to focus on decreasing BMI than for women.
- Given the positive association between higher levels of education and SE in both sexes, it is plausible that educational initiatives aimed at enhancing SE could be widely applicable. Nevertheless, given the more pronounced effect in men, this finding suggests that educational programs may work wonders in raising SE in this demographic.
- Particularly in women, the favorable link between smoking and SE emphasizes the need for focused anti-smoking initiatives addressing the alleged SE benefits of smoking. The development of more successful quitting programs depends on an awareness of the fundamental causes of smoking-related increased SE.
- Occupational health programs should include mental health and SE elements, as some jobs negatively affect SE and help reduce these impacts. Given the greater detrimental impact on women, tailoring these programs to meet their unique needs could be particularly beneficial.
- Given that men’s marital status has a negative effect on SE, it is possible that married men who are experiencing problems with their SE might benefit from marital therapy or support groups. Alternative support systems are necessary, as marital status has minimal impact on SE in females.
- Future studies should investigate these gender disparities further and consider additional factors, such as socioeconomic position and mental comorbidities, as potential factors that could impact the association between BMI and SE. Those persons who have a higher BMI can benefit from greater knowledge of these intricate relationships, which will help in the development of more effective measures to improve their SE and general well-being.

## Data Availability

The raw data supporting the conclusions of this article will be made available by the authors, without undue reservation.
